# MitoFish and MiFish Pipeline: A Mitochondrial Genome Database of Fish with an Analysis Pipeline for Environmental DNA Metabarcoding

**DOI:** 10.1093/molbev/msy074

**Published:** 2018-04-14

**Authors:** Yukuto Sato, Masaki Miya, Tsukasa Fukunaga, Tetsuya Sado, Wataru Iwasaki

**Affiliations:** 1 Center for Strategic Research Project, Organization for Research Promotion, University of the Ryukyus, Okinawa, Japan; 2 Department of Integrative Genomics, Tohoku Medial Megabank Organization, Tohoku University, Miyagi, Japan; 3 Department of Ecology and Environmental Sciences, Natural History Museum and Institute, Chiba, Chiba, Japan; 4 Department of Computational Biology and Medical Sciences, Graduate School of Frontier Sciences, The University of Tokyo, Chiba, Japan; 5 Department of Biological Sciences, Graduate School of Science, The University of Tokyo, Tokyo, Japan; 6 Center for Earth Surface System Dynamics, Atmosphere and Ocean Research Institute, The University of Tokyo, Chiba, Japan

**Keywords:** database, fish, mitochondrial genome, metabarcoding, environmental DNA

## Abstract

Fish mitochondrial genome (mitogenome) data form a fundamental basis for revealing vertebrate evolution and hydrosphere ecology. Here, we report recent functional updates of MitoFish, which is a database of fish mitogenomes with a precise annotation pipeline MitoAnnotator. Most importantly, we describe implementation of MiFish pipeline for metabarcoding analysis of fish mitochondrial environmental DNA, which is a fast-emerging and powerful technology in fish studies. MitoFish, MitoAnnotator, and MiFish pipeline constitute a key platform for studies of fish evolution, ecology, and conservation, and are freely available at http://mitofish.aori.u-tokyo.ac.jp/ (last accessed April 7th, 2018).

## Introduction

Fish occupy an important position in the vertebrate evolution and hydrosphere ecology, and genetic information from their mitochondrial genomes (mitogenomes) plays a key role in the investigation of their evolutionary histories and the protection and management of biological diversity. Mitofish is a database of fish mitogenomes with precise de novo annotations, and freely available at http://mitofish.aori.u-tokyo.ac.jp/. Since its major update in 2013 (Iwasaki et al. 2013), MitoFish has been actively and widely used by those in evolutionary science, ecology, ichthyology, fisheries, and conservation science from academia, government, and industry. MitoFish and its fish mitogenome annotation pipeline MitoAnnotator now receive >40,000 page views per year from around the world. In addition to its regular update of the data content, MitoFish has acquired two new functions since 2013. One is the multiple sequence annotation function, through which users can easily annotate many mitogenomic sequences for phylogeographic studies, for example.

The other, more important recent functional development in MitoFish is the implementation of MiFish pipeline (http://mitofish.aori.u-tokyo.ac.jp/mifish/). The recent advance in the high-throughput sequencing technology has enabled a new powerful approach in fish studies, that is, the metabarcoding analysis of environmental DNA (eDNA) (Deiner et al. 2017). It has been proved that fish (and tetrapod) mitochondrial DNA can be efficiently amplified by PCR from various environmental samples that include seawater, freshwater, sediment, and gut content (Miya et al. 2015; Ushio et al. 2017). eDNA analysis is a cost-effective and high-throughput approach to investigate species diversity in a noninvasive way, although several factors such as potential contaminations need to be taken cared of. MiFish is a set of universal PCR primers for effective metabarcoding of fish eDNA (Miya et al. 2015). As a powerful metabarcoding tool for biodiversity monitoring, MiFish primers were developed to target a hypervariable region within the fish mitochondrial 12S rRNA gene that is flanked by two highly conservative regions based on the MitoFish data.

MiFish pipeline on the MitoFish server is a user-friendly pipeline for analyzing fish metabarcoding data to estimate the species composition and ecological characteristics of natural environment. Whereas a number of computational tools are available for microbial metabarcoding analysis, there are few for the eDNA metabarcoding analysis of larger organisms. MiFish pipeline serves as a useful tool for those who are interested in diversity and ecological studies of fishes.

## MiFish Pipeline

**Fig. 1.**
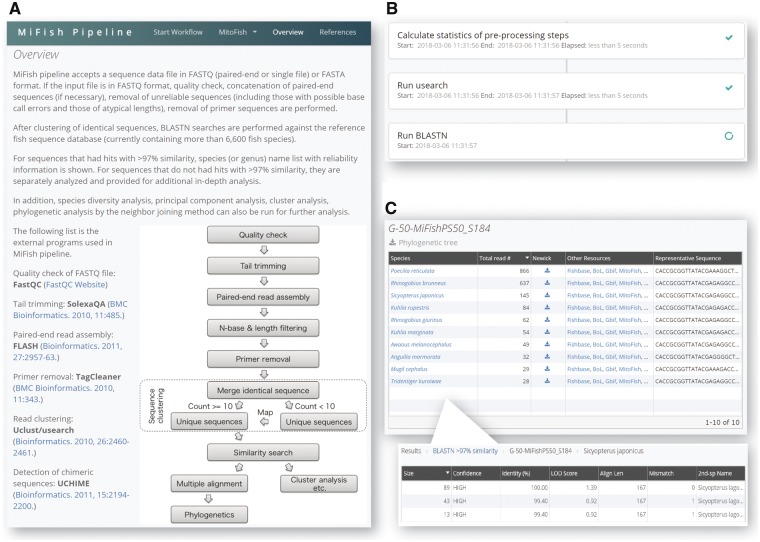
Screenshots of MiFish pipeline. (*A*) An overview of the pipeline. (*B*) A view of a progress status shown during pipeline execution. (*C*) An HTML report that contains results of the species assignment, sequence counts, and web-page links. For each species, a detailed report with confidence scores is provided.

As a new function on the MitoFish server, MiFish pipeline accepts and analyzes fish mitogenomic metabarcoding data, which include those produced using the MiFish primers (fig. 1*A*). The overall sequence quality is assessed by FastQC (http://www.bioinformatics.babraham.ac.uk/projects/fastqc/) and low-quality (Phred score < 10 by default) 3′-tails are trimmed by DynamicTirm.pl (Cox et al. 2010). The paired-end reads are merged by FLASH (Magoc and Salzberg 2011) and erroneous merged reads that contain N-nucleotides or do not have typical lengths are removed (229 ± 25 bp by default). The primer sequences are removed by TagCleaner (Schmieder et al. 2010) by allowing three-base mismatches at the maximum. Species-level taxonomic assignment is performed using Uclust (Edgar 2010) and NCBI Blast+ (Camacho et al. 2009; fig. 1*B*). Redundant sequences are merged into one sequence by keeping the count information. Then, low read-number sequences (<10 by default) are remapped onto high read-number sequences (≥10) at a given sequence-similarity threshold (99% by default), and the unmapped sequences are discarded. Blastn searches are conducted against MitoFish as a reference database with cutoff values of identity 97% and *e*-value 10^−5^, and species names of the top-hit sequences are retrieved. If the second to fifth top-hit sequences of each Blast search contain those of different species, confidence scores of the species assignment are calculated using the following formula:

$$\ln\frac{\left(\text{aligned length of the top}-\text{hit sequence}\right)/\left(\text{mismatch numbers of the top}-\text{hit sequence}+1\right)}{\left(\text{aligned length of the second}-\text{hit sequence}\right)/\left(\text{mismatch numbers of the second}-\text{hit sequence}+1\right)}$$

All-species and within-species molecular phylogenetic trees are estimated for each environmental sample. Multiple sequence alignments are generated by MAFFT (Katoh and Standley 2013) and neighbor-joining phylogenetic trees are estimated by Morphy (Adachi and Hasegawa 1992). An HTML report is finally presented, which can also be used for calculating ecological indices such as alpha diversity, beta diversity, and correlation coefficients (fig. 1*C*). This report also contains links to major databases such as FishBase (Froese and Pauly 2017), Barcode of Life (Ratnasingham and Hebert 2007), Global Biodiversity Information Facility (Edwards 2004), and MitoFish.

**Fig. 2.**
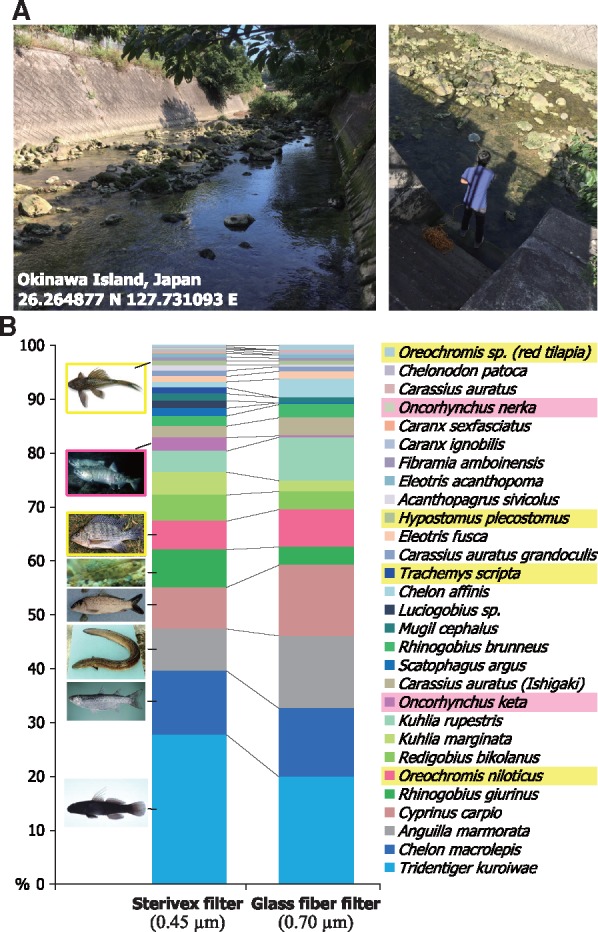
Application of MiFish pipeline to a fish eDNA data set. (*A*) Photos of the sampling site in Okinawa Island, Japan. (*B*) A summary of the species assignment results produced by MiFish pipeline. The left and right bars indicate the data of eDNA extracted using the Sterivex and glass-fiber filters, respectively. Read numbers are expressed as percentages to the total read numbers. Nonnative species in Okinawa Island are highlighted (Yellow: Invasive species, Pink: Salmons). Fish pictures are from FishBase.

Figure 2 shows an example of fish eDNA analysis results produced by MiFish pipeline. The water sample was taken at Uchidomari river in Okinawa Island, Japan (fig. 2*A*) and filtrated up to 960 ml using 0.45 µm Sterivex filter (Millipore) or 0.70 µm glass-fiber filter (Whatman GF/F). DNA was extracted using DNeasy PowerWater Sterivex and DNeasy Blood & Tissue kits (Qiagen) from the Sterivex and glass-fiber filters, respectively. MiFish primers (Miya et al. 2015) were used to amplify eDNA with the annealing temperature of 60°C. MiSeq with V2 chemistry (Illumina) was used for 150-bp paired-end sequencing.

The estimated species composition well represented the fish community in the rivers in Okinawa Island (fig. 2*B*). Dominant species included those typically observed in Okinawa Island rivers such as gobies (e.g., *Tridentiger kuroiwae*, *Rhinogobius giurinus*, and *Redigobius bikolanus*), mullets (e.g., *Chelon macrolepis* and *Chelon affinis*), and giant mottled eel (*Anguilla marmorata*), as well as invasive alien species such as nonnative tilapias (genus *Oreochromis*) and plecos (*Hypostomus plecostomus*). It may be noted that salmons (genus *Onchorhynchus*) also appeared in the list presumably because they are widely consumed as food in Okinawa and drainage likely contains their eDNA. These results would exemplify that MiFish pipeline is a useful tool for eDNA analysis of the endemic fish species composition, invasive species, and also impact of human activities, whereas the detection of salmons also suggests that eDNA analysis can be affected by unexpected environmental influences and/or contamination and need to be interpreted with caution. Taken together, MitoFish, MitoAnnotator, and MiFish pipeline constitute a key platform for studies of evolution, ecology, and conservation of fishes.
